# Current SIDS research: time to resolve conflicting research hypotheses and collaborate

**DOI:** 10.1038/s41390-023-02611-4

**Published:** 2023-05-12

**Authors:** Paul N. Goldwater

**Affiliations:** Adelaide Medical School, Faculty of Health and Medical Sciences, The University of Adelaide, North Terrace, Adelaide, SA Australia

## Abstract

**Abstract:**

From the earliest publications on cot death or sudden infant death syndrome (SIDS) through to this day, clinical pathology and epidemiology have strongly featured infection as a constant association. Despite mounting evidence of the role of viruses and common toxigenic bacteria in the pathogenesis of SIDS, a growing school of thought featuring a paradigm based on the triple risk hypothesis that encompasses vulnerability through deranged homoeostatic control of arousal and/or cardiorespiratory function has become the mainstream view and now dominates SIDS research. The mainstream hypothesis rarely acknowledges the role of infection despite its notional potential role as a cofactor in the triple hit idea. Decades of mainstream research that has focussed on central nervous system homoeostatic mechanisms of arousal, cardiorespiratory control and abnormal neurotransmission has not been able to provide consistent answers to the SIDS enigma. This paper examines the disparity between these two schools of thought and calls for a collaborative approach.

**Impact:**

- The popular research hypothesis explaining sudden infant death syndrome features the triple risk hypothesis with central nervous system homoeostatic mechanisms controlling arousal and cardiorespiratory function. Intense investigation has not yielded convincing results. There is a necessity to consider other plausible hypotheses (e.g., common bacterial toxin hypothesis).
- The review scrutinises the triple risk hypothesis and CNS control of cardiorespiratory function and arousal and reveals its flaws.
- Infection-based hypotheses with their strong SIDS risk factor associations are reviewed in a new context.

## Introduction

There are two leading research hypotheses used to explain sudden infant death syndrome (SIDS). The mainstream popular research hypothesis features the triple risk hypothesis^1^ with central nervous system (CNS) homoeostatic mechanisms controlling arousal and cardiorespiratory function and invokes prone sleep position as playing a causal role.^2^ The other is the common bacterial toxin hypothesis,^3–5^ which utilises experimental and epidemiological evidence indicating viral infection combined with bacterial toxaemia and prone positioning may produce a fatal outcome through super antigenic shock. The review scrutinises these hypotheses and suggests a different way forward.

## The common bacterial infection hypothesis

From the earliest epidemiological studies on cot death or as it was later defined^6–8^ as SIDS, there were clear indications that infection, especially respiratory viral, was associated with these deaths.^9–13^ The common bacterial toxin hypothesis was developed on the basis that a viral infection (along with prone positioning) induced upper respiratory tract changes conducive to toxin production by toxigenic bacteria (including *Staphylococcus aureus, Streptococcus pyogenes* and *Escherichia coli*), all of which were commonly found to colonise the nasopharynx.^14–16^ In >50% of cases, Staphylococcal toxins were demonstrated in SIDS babies’ tissues.^17–20^ These were identified in tissues of 33/62 (53%) SIDS infants from three different countries: Scotland (10/19, 56%); France (7/13, 55%); Australia (16/30, 53%). In the Australian series, toxins were identified in only 3/19 (16%) non-SIDS deaths (*χ*^2^ = 5.42, *P* < 0.02).^17^ Harrison et al.^18^ demonstrated that sleeping prone caused pooling of secretions and increased numbers of toxigenic bacteria in the nasopharynx and Malony et al.^19^ showed prone sleeping increased the local temperature into ranges known to induce bacterial toxin production.^19^

The hypothesis suggested viral infection acted as a trigger for events leading to super antigenic toxic shock through T-cell activation by staphylococcal enterotoxins or toxic shock syndrome toxin-1. Staphylococcal enterotoxin-*like* proteins also act as superantigens.^20^ and could also be involved in SIDS. A mouse model developed by Nobel Laureate Peter Doherty and colleagues showed that mice infected with the respiratory zoonotic pathogen lymphocytic choriomeningitis virus (LCMV) were unharmed, but in virally infected mice given an intraperitoneal injection of Staphylococcal enterotoxin B, this was rapidly lethal. Staphylococcal toxin injection alone was non-lethal.^21^

The respiratory tract in SIDS frequently shows evidence of inflammatory involvement of the airways and lungs.^11,22,23^ The inflammatory process may involve platelet aggregation and obstruction of the lung capillaries by blood platelet aggregates and leucocytes.^24^ This could provide clues to the pathogenesis of intrathoracic petechial haemorrhages observed in 80–90% of SIDS cases. Intrathoracic petechial haemorrhages have been explained by mainstream researchers as resulting from agonal changes in intrathoracic pressure.^25^ Animal experimentation has failed to affirm this idea.^26^

My interest in SIDS research was aroused through my colleague, the late Dr Karl A. Bettelheim who had demonstrated in a paper given at a meeting in Auckland in the early 1980s that sera obtained from cases of SIDS was lethal to infant mice upon intraperitoneal injection. Whether the mice were also congenitally infected with an enzootic virus was not at the time a consideration. Karl had published widely on *E. coli* and human infant disease. Knowledge of the various toxins of *E. coli* and the common finding of the bacterium in the respiratory tract of SIDS babies led us to investigate the possible role of *E. coli* in SIDS. Interesting but inconclusive correlations were found.^27,28^

As mentioned, *S. aureus* is also commonly found in the upper and lower respiratory tract of SIDS cases.^18,29^ Significantly greater proportions of SIDS compared with control/comparison babies were positive for *S. aureus* (68.4% vs. 40.5%) and for staphylococcal enterotoxin genes (43.8% vs. 21.5%), suggesting a possible role in SIDS.^30^

The further analysis enabled us to demonstrate a significant relationship between colonisation with *S. aureus* and the risk factor of prone sleep position in SIDS.^31^ The work showed numerous combinations of the nine enterotoxins in the cases of SIDS. However, the DNA extracts used in the Highet et al. study^31^ were re-examined using an lllumina MiSeq platform by Leong et al.^32^ In this study, the frequency of detection of *S. aureus* did not differ significantly from the comparison babies.^32^ We explain the disparity between the studies on methodological differences.

Derived from the staphylococcal enterotoxin study,^30^ we proposed that contamination of the baby’s sleeping surface with *S. aureus* might explain the relationship with prone sleeping, given that potentially contaminated sleeping surfaces such as the parental bed,^33^ sofa,^33,34^ and used cot mattresses^35^ were established risk factors for SIDS.

The idea that prone positioning in relation to SIDS could affect the vagus nerve^36^ and its multitudinous functions, including influence on the gut microbiota, on gut hormones and the cholinergic anti-inflammatory pathway, were based on the vagus nerve inflammatory reflex, known to prevent cytokine-induced tissue damage and death. Vagal stimulation in animal models prevents cytokine release and damage during sepsis, shock, endotoxemia, etc. Prone positioning may affect vagal neurophysiology adversely. This subject remains unexplored in the context of SIDS.

## Reappraisal of the popular mainstream SIDS research hypothesis

The triple risk hypothesis^1^ formed the basis for hypotheses centred on the CNS/brainstem control of arousal, respiration, and cardiac function as well as a focus on the prone sleep position and the sleeping environment.^2^ The paradigm explains prone sleep position as playing a causal role;^37^ this seems disingenuous given that babies die in supine and side positions which should necessarily dictate different mechanisms of demise. Rather, it would be logical to consider a prone sleep position increasing the risk of SIDS through an unknown mechanism. Airway obstruction in prone sleepers would make it implausible to attribute non-prone SIDS deaths to a similar mechanism. An explanation may reside in an increased risk in prone over other positions. As alluded to previously, such increased risk could relate to prone sleep position increasing the likelihood of colonisation by toxigenic bacteria from the sleeping surface and the increased likelihood of induction of bacterial lethal toxins. This is discussed further below.

In a different context, the attribution of sleep position with causality has led to an argument for a causal relationship between supine sleep position and autism spectrum disorder; based on the increase in autism rates following the introduction of the Back-to-Sleep (BTS)/Reducing-the-Risk (RTR) campaign in five different countries.^38^ Association does not equal causation.

## Neuropathology and SIDS

In 1990, Oehmichen^39^ described the state of SIDS neuropathological research as ‘Due to differences in the findings as well as methodologic and interpretative problems, no definitive *pathogenetic concept* based on the available neuropathologic findings can be formulated at present, even though many observations tend to indicate that the brainstem, as the central organ controlling respiration, is probably of prime importance in SIDS. Even the classification of the described phenomena as primary and secondary changes can be and is disputed. No diagnostic criteria for classification of SIDS and control cases could be established, since all obtained criteria are nonspecific, and the described criteria are not present in all SIDS cases’. Two decades on and the same message applies with the possible role of the CNS in SIDS remaining confused. Findings involving neurotransmitters (e.g., 5HT, its receptors and gene polymorphisms)^40^ have not led to conclusive results. While hypoxic-ischaemic neuronal injury (and neuronal apoptosis) is generally thought to be common in SIDS cases,^41–43^ none of the authors have considered a role for sepsis in these processes. Sepsis is an established leading cause of hypoxia/ischaemia and neuronal apoptosis.^44,45^

The researchers consider that the described neuropathology is a primary phenomenon and have rarely considered that these changes could be the result of a secondary effect, say, from cytokine responses to viral infection or effects of bacterial toxaemia/super antigenic shock. Many of the CNS findings seen in SIDS cases are also observed in control babies.^46^ In rare attempts to correlate CNS findings with epidemiological risk factors have not resulted in substantial success. Examples of such correlation include male sex and age for a restrictive pattern of neuropathological findings.^47^ On the other hand, Duncan et al.^43^ found no male gender relationship with various neuropathological/neurotransmitter findings in SIDS brains.^43^ Suffice to say, the role of infection in SIDS has been largely ignored by mainstream researchers.

## Explaining the prone position risk factor

Blackwell et al.^48^ and Goldwater^49,50^ listed the genetic, developmental and environmental SIDS risk factors, all indicating susceptibility to infection. This list, with some modifications, is shown in Table 1. This information might help convince researchers of the importance of infection in SIDS.

**Table 1:** Risk factors for SIDS that parallel risk factors for susceptibility and/or relationship to infection.

Genetic
Ethnicity
Male sex
Developmental
Prematurity/Intrauterine growth retardation
Night time deaths
Peak age range 2–4 months
Prenatal/pregnancy
Higher parity
Low birth weight, short gestation (intrauterine growth retardation)
Inadequate prenatal care
Maternal smoking
Environmental
Mild infections (URTI or gastroenteritis) (recent illness potentiates effect of prone sleep position and overwrapping)
Recent visit to general practitioner or outpatient clinic
Prone sleeping
Cigarette smoke exposure
Overheating
Cooler season
Lack of breastfeeding
Poor socio-economic conditions
No or late immunisation
Air pollution
Contaminated sleeping surface: used cot mattress, sofa, parental bed
Daycare attendance
High birth order/older siblings

For references, see refs. ^48–50^

A convincing explanation of the risk factor of prone sleep position has not been achieved by the mainstream. There is, however, a compelling explanation provided in two well-designed and independent, geographically disparate epidemiological studies (Tasmanian^51^ and Scandinavian^52^) that link infection (with prone sleep position) to SIDS. In the Tasmanian study, infection and prone sleep position featured strongly: the study revealed a 10-fold increased risk of SIDS if prone-sleeping babies were ill with features of an infection, but it was associated with only a slight increase in risk among infants considered well. The Scandinavian study revealed a 29-fold increase in risk if prone-sleeping babies had an infection. Both studies showed that exposure to cigarette smoke increased the risk of SIDS. Smoke and infection combine with lethal consequences: in general, bacterial and viral infections can be synergistic^53,54^ and both are exacerbated by exposure to smoke.^55^

There are laboratory findings on SIDS which point to the underlying infection. These are set out in Table 2.

**Table 2:** Laboratory findings in SIDS cases (for references see refs. ^48–50^).

Mild acute inflammatory changes in airways, lungs, and myocardium
Proteomic and immunohistochemical evidence of infection and responses to infection bacterial toxins in tissues
IgG response to bacterial toxins
Increased IgM response to core endotoxin
Increased levels of mast cell tryptase
Increased levels of mannose-binding lectin
Raised fibrin degradation products
CD68 immunoreactivity in airways and brain
Raised IL-6 in vitreous humour, cerebrospinal fluid, and liver
CSF lymphocytosis
Presence of tracheal/lung IgM
IL-10 low producer
IL-1b high producer
Raised IgA in duodenum and saliva
Normally sterile site cultures yielding a bacterial pathogen

## Prone sleep position and the Back-to-Sleep/Reducing-the-Risks campaigns

The BTS and RTR campaigns have drawn some of their success from an anomaly of how SIDS deaths were recorded in the 1970s, 1980s and 1990s. There is compelling evidence of diagnostic shifting during those decades resulting in a possible exaggerated rise in SIDS numbers in the 1980s and a complimentary fall in the 1990s.^56–62^ The introduction of new infant vaccines in 1990 could possibly have contributed. The apparent relationship between the BTS/RTR campaigns and the reduction in SIDS deaths has not been subjected to rigorous scientific scrutiny. Assumptions have been accepted without question. This is not to say that putting babies on their backs to sleep has not had beneficial effects. However, the effect of supine sleeping in the USA and several other countries has plateaued and SIDS numbers remain unacceptably high.^63^ Moreover, SIDS deaths significantly increased between 2019 and 2020.^64^ It is yet to be determined whether SARS-Cov-2 virus played a role.

SIDS is largely a disease of poverty, poor hygiene, overcrowding, prematurity, exposure to smoke in pregnancy and postnatally. These are features common to many transmissible infectious diseases. Sleeping prone on second-hand mattresses,^33^ the parental bed,^31^ or sofa^32^ (contaminated surfaces) increases the risk of SIDS, as do male sex^65^ and high birth order with older siblings bringing viral infection home.^65^ SIDS is more frequent in rural areas^66^ and tends to occur more frequently in winter.^67,68^ These facts should alert us to the possibility of an epizootic agent playing a role, in addition to seasonal respiratory viruses. LCMV would fit well here.^69^ As mentioned, a convincing SIDS animal model has been demonstrated with this virus.^19^

## Conclusion

All research should be founded on logical and scientifically plausible constructs. Without these, a successful conclusion would be impossible. The apparent lack of progress in determining a cause or causes of SIDS (despite the help of twenty-first-century science and technology) should call for a reappraisal of the fundamental mainstream hypotheses.

SIDS research is encumbered with unusual limitations;^70^ these include ethical issues regarding consent for obtaining and retaining tissue, and the problem of difficulty in obtaining suitable control material for meaningful research. Notwithstanding these, infection, a key pointer in the SIDS story, has been largely ignored by mainstream research or given minimal attention. Few, if any, of the key infection-related papers on SIDS mentioned above are ever cited in mainstream papers. Is this citation amnesia^71^ or the ‘disregard syndrome?’^72^ Both are well described in many areas of scientific research and are counterproductive and unethical. The basis of this failure to acknowledge established evidence of the role of infection in SIDS is difficult to understand, but its origins are likely to involve the politics of research grant funding and restrictive thinking. Continuation of such a narrowed approach will delay the explanation of the tragic enigma of SIDS. It is surely time to reconsider and collaborate. The items listed in Tables 1 and 2 provide fertile ground upon which to develop productive research outcomes. The overwhelming number of infection-related factors, including risk factors (age, sex, immunity, smoke exposure, seasonality, rural preponderance, etc.), would surely invite serious investigation. Using contemporary application of Koch’s postulates^73^ interpretation of key infection-related findings such as staphylococcal toxins in SIDS tissues^9–15^ (especially when these are found in cases from three different geographical regions^15^) would, on the evidence, be regarded by infectious diseases experts as ‘the main cause of death’ in babies meeting the SIDS definition. Paradoxically, if a multidisciplinary death review panel agreed that a staphylococcal toxin was the cause of death, then, based on the Bajanowski et al. recommendations,^20^ the case would then be classified as an explained infant death. It is reasonable to ask why the staphylococcal toxin findings^9–15^ in more than 50% of cases have been ignored for so long and that routine testing for these toxins had not been widely applied by those responsible for investigating sudden unexpected infant deaths? Given the findings of this review, a way forward could benefit from a broader collaborative approach to this singularly challenging task.
